# Detection and genotypes of *Toxoplasma gondii* DNA in feces of domestic cats in Colombia

**DOI:** 10.1051/parasite/2020023

**Published:** 2020-04-17

**Authors:** Alejandro Zamora-Vélez, Jessica Triviño, Sebastián Cuadrado-Ríos, Fabiana Lora-Suarez, Jorge Enrique Gómez-Marín

**Affiliations:** 1 Grupo de Estudio en Parasitología y Micología Molecular (GEPAMOL), Centro de Investigaciones Biomédicas, Facultad Ciencias de la Salud, Universidad del Quindío 630004 Armenia Colombia; 2 Grupo de Biodiversidad y Conservación Genética, Instituto de Genética, Universidad Nacional de Colombia 111321 Bogotá Colombia

**Keywords:** *Toxoplasma gondii*, Cats, PCR, Prevalence, *ROP18*

## Abstract

The high prevalence of *Toxoplasma gondii* in the human population in Colombia has been linked to the existence of a high density of urban stray cats, exposing the whole population to a high density of oocysts. The goal of this study was to determine the DNA prevalence of *T. gondii* by conventional PCR and to phylogenetically analyze ROP18 sequences from positive samples in domestic cat (*Felis catus*) fecal samples in the city of Armenia, Quindío. Fecal samples from 140 cats were collected from 10 districts around the city. Samples were concentrated using Ritchie’s method and analyzed through optical microscopy. Concentrates were used for DNA extraction followed by nested PCR amplification for *T. gondii* gene B1. PCR for ROP18 was performed on all B1 positive samples; the ROP18 sequences obtained were related to the Archetype I Brazilian and Chinese strains. No oocysts were detected by optical microscopy; however, 17.8% (25/140) B1 and 24% (6/25) ROP18 PCR-positive samples were detected. Phylogenetic analyses showed that isolates clustered into a single group. We assessed whether associations existed between *T. gondii* positive fecal samples and survey variables such as cat healthcare and socioeconomic characteristics of owners, but no statistically significant associations were found. The presence of *T. gondii* in cat feces is an important factor contributing to the high prevalence in the human population of this city.

## Abstract (fr)

La forte prévalence de *Toxoplasma gondii* dans la population humaine en Colombie a été liée à l’existence d’une forte densité de chats errants urbains, exposant l’ensemble de la population à une forte densité d’oocystes. Le but de ce travail était de déterminer la prévalence de l’ADN de *T. gondii* par PCR conventionnelle et d’analyser phylogénétiquement les séquences ROP18 d’échantillons positifs dans des échantillons fécaux de chat domestique (*Felis catus*) dans la ville d’Armenia, Quindío. Des échantillons fécaux de 140 chats ont été collectés dans 10 districts de la ville. Les échantillons ont été concentrés en utilisant la méthode de Ritchie et analysés par microscopie optique. Des concentrés ont été utilisés pour l’extraction d’ADN suivie d’une amplification par PCR nichée pour le gène B1 de *T. gondii*. La PCR pour ROP18 a été réalisée sur tous les échantillons positifs pour B1 ; les séquences ROP18 obtenues étaient apparentées aux souches Archétype I brésiliennes et chinoises. Aucun oocyste n’a été détecté par microscopie optique mais les échantillons étaient positifs par PCR pour 17,8 % (25/140) pour B1 et 24 % (6/25) pour ROP18. Les analyses phylogénétiques ont montré que les isolats formaient un seul groupe. Nous avons évalué s’il existait des associations entre des échantillons fécaux positifs à *T. gondii* et des variables d’enquête telles que les soins de santé des chats et les caractéristiques socioéconomiques des propriétaires, mais aucune association statistiquement significative n’a été trouvée. La présence de *T. gondii* dans les excréments de chats est un facteur important contribuant à la forte prévalence dans la population humaine de cette ville.

## Introduction

*Toxoplasma gondii* is an obligate intracellular parasite with worldwide distribution inducing toxoplasmosis and infecting humans via warm-blooded animals. Wild and domestic felids are the only known definitive hosts with the ability to shed oocysts in their feces. Common pathways of infection include oocyst-contaminated water, soil, and food; tissue cysts in undercooked or raw meat; and congenital transmission [13, 20, 37, 43]. Oocysts are the environmentally resistant form of the parasite and play a key role in transmission to new hosts and ecosystems, generating the need to study humans alongside domestic and wild animal populations [43]. The large number of oocysts shed during primary-infection by felids could lead to extensive environmental contamination, which can infect a high number of intermediate species, such as humans, mice or birds. [26]. High rainfall rates can facilitate survival of oocysts for months, explaining why regions with higher precipitation show higher prevalence compared to arid regions, which show far lower rates of infection in the population living in these areas [1, 16, 25].

In Colombia, the prevalence in the human population varies between 30% and 60% [6], and this high prevalence has been linked to the existence of a high density of urban stray cats, exposing people to an elevated density of oocysts [10]. This high density of free-ranging domestic cats can explain why cat ownership in homes does not increase the risk for *T. gondii* in surveys in some cities in Colombia [29]. A study in Armenia city in 1998 found 89.3% seroprevalence in 28 domestic cats and detected a 66.6% shedding prevalence of *T. gondii*-like oocysts by microscopy in fecal samples from 18 cats [30]. In 2006, another study found a seroprevalence of 84.8% in 33 stray cats, but no oocysts were identified by microscopy in feces collected from the rectum from these cats [10]. Although training helps with identification of *T. gondii*-like oocysts, morphological structure along cannot confirm that the oocysts visualized are actually *T. gondii*, as *Hammondia* oocysts for example look identical [11]. Consequently, molecular detection-based methods, like PCR, can be an alternative and complementary method to microscopy to identify cats infected with *T. gondii.* As a consequence, the objective of this study was to determine the prevalence of *T. gondii* DNA in cat fecal samples by conventional PCR and to analyze the *ROP18* gene from positive samples in Armenia, Quindío, Colombia.

## Methodology

### Sample

Armenia is the capital of the department of Quindío in Colombia’s central mountain range, at an altitude of 1480 m above sea level. Armenia has 301,226 inhabitants, according to the projections of the Administrative Department for National Statistics (DANE, 2018). Armenia is divided into 10 administrative zones, called communes. The number of cats to sample was estimated according to the rabies vaccination program statistics reported in Armenia, with a population of 15,015 owned cats [28]. Therefore, considering inferences with prevalence with 95% confidence, an accepted error margin of 8%, and using an expected prevalence of 66.6% based on previous studies, at least 132 owned cats needed to be sampled according to Epiinfo v.7.2.3.1 software. The residences were selected at random in the 10 communes of the city of Armenia.

### Questionnaire

The owners were interviewed about the following aspects: number of stray cats observed 500 m around the residence; commune; age and sex of domestic cats; kind of food that the domestic cats eat; water source for domestic cats to drink; last deworming (recently: 30 days ago or less; not long since: 30–90 days; long ago: more than 90 days); and hunting habits.

### Microscopic analysis

The cat feces samples were processed with the Ritchie technique [35], approximately 5 g of fecal sample from each cat were diluted in 15 mL of 0.9% sterile saline solution and centrifuged for 5 min at 1600 ×*g*. This washing procedure was repeated three times. We discarded the supernatant, and resuspended the pellet using 5 mL of sterile saline solution. Then, we added 5 mL of 10% formalin solution and 3 mL of 99% diethyl ether (Sigma, USA) to each tube. We sealed and rigorously shook the tube to bring the diethyl ether into contact with all parts of the sediment and performed a new centrifugation at 1000 ×*g* for 2 min. After this, 30 μL of sediment were scanned in triplicate using Olympus microscopy with 40× objective lens using 1% parasitological lugol (Químicos Albor, Colombia) as a contrast visual. The microorganisms were identified by three observers and confirmed with the collaboration of Dr. Fidel Angel Nuñez from the Tropical Medicine Institute Pedro Kouri, Havana, Cuba.

### *Toxoplasma gondii* DNA extraction procedure and PCR detection

The resultant pellet from the Ritchie technique was used to obtain DNA from cat feces. The pellets were washed four times in 2 mL tubes with PBS and centrifuged at 4500 ×*g* for 10 min. The supernatant was discarded and 600 μL of DNAzol (Invitrogen, USA), 10 μL of isoamyl alcohol (Fisher Scientific, USA) and 0.3 g of zirconium silicate beads with 0.5 mm diameter (BioSpec, USA) were added. Afterwards, the tubes were shaken five times in a Mini Bead Beater (BioSpec, USA) to maximum speed for 1 minute and 1 minute in ice [41]. Later, a Wizard Genomic DNA extraction kit (Promega, USA) was used for nuclear lysis and purification, following the manufacturer’s protocol. In order to detect DNA, the *T. gondii B1* repetitive fragment (GenBank accession number AF179871) was used in an amplification nested PCR method [5]. All B1 positive samples were sequenced to confirm that the amplified product was in fact *T. gondii*, because it is possible to amplify DNA from other organisms [27]. As has been previously described [31, 41, 45], the primers for the first PCR were Toxo N1 5′–GGAACTGCATCCGTTCATGAG–3′ and Toxo C1 5′–TCTTTAAAGCGTTCGTGGTC–3′ to obtain a 193-bp fragment. The second PCR was performed with the primers Toxo N2 5′–TGCATAGGTTGCCAGTCACTG–3′ and Toxo C2 5′–GGCGACCAATCTGCGAATACACC–3′ to obtain a 96-bp fragment. The first amplification protocol consisted of one initial stage of denaturation for 5 min at 94 °C, followed by 40 cycles of amplification, and 1 cycle consisting of 1 min at 94 °C for DNA denaturation, 1 min of annealing at 53 °C, and 1 min of extension at 72 °C. Subsequently, an additional step of 10 min of final extension at 72 °C was performed. The second PCR with the product of the first amplification, consisted of one initial stage of denaturation for 5 min at 94 °C, followed by 14 cycles of amplification, and 1 cycle consisting of 1 min at 94 °C for DNA denaturation, 1 min of annealing at 53 °C, and extension at 72 °C for 30 s. Then, an additional step of 10 min of final extension at 72 °C was carried out. The *ROP18 T. gondii* sequence (GenBank accession number JX045319.1) was detected in *B1* PCR-positive samples. To detect this sequence, we used forward primer ROP18S 5′–GACCGTCTTTCAAGAGGAGGA–3′ and reverse primer ROP18R 5′–ACGCTGGTGAGAGGTGCAC–3′ to obtain a 514-bp fragment. The amplified protocol consisted of one initial stage of denaturation for 3 min at 94 °C followed by 35 cycles of 30 s at 94 °C for DNA denaturation, 45 s at 60 °C for annealing, and 30 s at 72 °C for extension. Then, 5 min at 72 °C for final extension was performed. All primers were synthetized by Invitrogen Corporation (USA). Finally, a 1.5% agarose electrophoresis gel was used to analyze PCR products. The positive control was DNA from the *T. gondii* control RH strain, and the negative control was distilled water in the presence of primers.

### Statistical analysis

As the dependent variable was binary (presence vs*.* absence of *T. gondii* DNA in fecal samples), a Chi-squared test was used to assess relationships with other variables including demographic, behavioral and cat care. A *p* value < 0.05 for the significance level was employed. OR values were calculated with a 95% confidence interval. The Statgraphics Centurion v.17 software was used.

### Sequencing and phylogenetic analyses

PCR-positive products for *ROP18* were gel-purified from low-melt agarose gels, followed by recovery using a Wizard PCR SV and PCR clean up system kit (Promega, USA). Sequencing was done under BigDye^®^ terminator cycling conditions by using the normal automatic service by Macrogen (South Korea) in a 3730XL DNA sequencer with the same primers as the PCR amplifications. BLAST (http://blast.ncbi.nml.nih.gov/Blast.cgi) search on the GenBank database with all *ROP18* sequences was performed, verifying that they belong to *T. gondii*, as well as to identify closely related genotyped strains. We explored strain relatedness in more detail through phylogenetic analyses, including *ROP18* sequences available in GenBank and ToxoDB. In a previous study*, T. gondii* ROP16 sequences from human and meat samples obtained in the same study area showed a high degree of genetic divergence and clustered with highly virulent strains, and we expected the *ROP18* sequences we obtained to show the same pattern within the phylogeny [2]. Sequences were aligned with *T. gondii* sequences available in GenBank and ToxoDB (Supp. Table 1), restricting the number of sequences per strain to one (except for specific strains with sequences in both GenBank and ToxoDB). We included *ROP18* sequences isolated from patients with ocular toxoplasmosis in the same study area [38]. Alignment was performed with MAFFT v7.187 [21], using the auto routine and default settings. Most of downloaded sequences corresponded to the complete gene (1663–1671 bp), depending on the strain [15], and sequences obtained here represented only a partial region. Therefore, we decided not to trim the resulting alignment, as regions dominated by gaps, insertions or deletions usually contain phylogenetically useful information, and the percentage of identity obtained in BLAST suggested that our sequences were sufficiently informative.

The evolutionary model and partition scheme that best suited the aligned sequences were determined using PARTITIONFINDER v 1.1.1 [22], according to the corrected Akaike information criterion (AICc). The optimal partitioning was the minimum partitioning scheme, and the resulting model was K80 [39]. These parameters were implemented in the Bayesian analyses executed in BEAST 1.8.3 [8]. We assumed a constant population size to estimate the patterns of speciation across the phylogeny, and a strict clock; we kept priors at the default. The number of Markov chain Monte Carlo (MCMC) iterations was set to 100 million, with 25% of trees obtained as burning, retaining trees each 1000 steps. Two independent chains were run and we checked effective sample size (ESS) in Tracer v1.6 [8]. The maximum clade credibility (MCC) tree was estimated in TreeAnnotator [8], discarding 25% of initial trees. We also investigated alternative phylogenetic grouping, constructing an unrooted network (split network) with the Neighbor-Net method implemented in SplitsTree4 [18]. We estimated a haplotype network, with equal weighting on transversions/transitions, without considering gaps/missing and removing invariable sites, and the median-joining network algorithm [3] implemented in PopART [24].

## Results

### Prevalence of *Toxoplasma gondii* by microscopy and PCR in cat fecal samples

**Figure 1:**
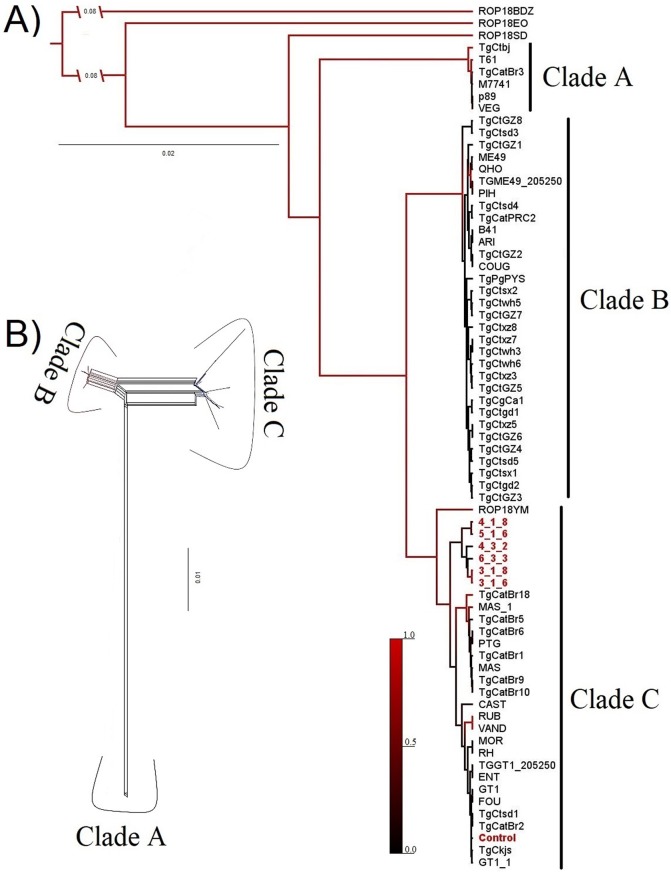
(A) Phylogenetic relationships among *ROP18* sequences from several strains of *T. gondii* as reported in GenBank, and sequences found in cats in Armenia in present study are shown with numbers (6_3_3; 5_1_6; 3_1_6; 3_1_8; 4_3_2; 4_1_8) and in red font. The phylogeny was inferred by Bayesian analysis in BEAST. Branch lengths indicate genetic distance, and posterior probabilities of nodes are represented by the size of the branches that separate from the node. Major clades labeled are supported by absolute support (PP < 0.99). (B) Neighbor-net phylogenetic network based on the rhoptry protein 18 gene from *T. gondii* strains. Potential reticulation between strains is denoted by the network patterns connecting strains.

Between November 2014 and August 2015, in the communes selected, 521 residences were visited. In 217 of these houses (41.6%), there was at least one domestic cat, and in 140 of the 217 houses (64.5% of visited houses with at least one domestic cat), it was possible to collect fecal samples with permission from the owners. We found via optical microscopy, *Ancylostoma* spp. in 6/140 samples (4.3%), *Toxocara* spp. in 6/140 samples (4.3%), *Toxascaris* spp. in 3/140 samples (2.1%), and *Hymenolepis* spp. in 4/140 samples (2.8%) (Fig. 1). *Toxoplasma gondii*-like oocysts were not observed.

Of the 140 collected samples, 25 (17.85%) were positive for *T. gondii* DNA by B1 PCR and confirmed through sequencing according to the results obtained from Macrogen (South Korea). Prevalence among the communes varied between 0% and 37.8% (Table 1), but these differences did not attain statistical significance (χ^2^ test *p* = 0.27).

**Table 1:** Distribution of the samples collected in Armenia, Quindío, Colombia during 2014–2015 by commune and positive samples from domestic cat feces using *B1* PCR to detect *T. gondii* DNA.

Commune	Number of samples	Number of positive samples	% positive samples	Confidence interval (95%)
1	20	2	10	[7.6–12.3]
2	17	1	5.9	[4.6–7.1]
3	17	3	17.6	[15.1–20.0]
4	14	4	28.5	[24.4–32.5]
5	19	7	36.8	[33.7–39.8]
6	10	1	10	[5.4–14.5]
7	18	3	16.6	[13.9–19.3]
8	12	1	8.3	[5.1–11.4]
9	8	3	37.8	[31.8–43.7]
10	5	0	0	0

In all, 10/56 positive samples (17.8%) were from male cats and 15/78 (19.2%) were from female cats. Also, 6/45 positive samples (13.3%) were from young cats (<1 years old), 15/68 (22%) were from adult cats (between 1 and 7 years old), 2/12 (16.6%) from older cats (>7 years old), and 2/15 (13.3%) were from cats without identify age data (Table 2). The 7 years used as an age cut-off was a reference from Hand et al., 2000 [17]. There were no significant associations between the data obtained from the questionnaire and the prevalence of *T. gondii* DNA-positive samples through Chi squared and OR tests (Table 3).

**Table 2:** Comparison of PCR *T. gondii* prevalence in cat feces according sex and age (years) in the 140 cats from which the samples were taken in 10 communes from Armenia.

Cats (140)	*B1* PCR
(*N*; %)	Positive samples/*N* (%)
Gender (140; 100%)	
Female (78; 55.7%)	15/78 (19.2%)
Male (56; 40%)	10/56 (17.8%)
Non-determined (6; 4.3%)	0 (0%)
Age in years (140; 100%)	
≤ 1 (45; 32.14%)	6/45 (13.3%)
> 1 y ≤ 7 (68; 48.5%)	15/68 (22%)
> 7 (12; 8.5%)	2 /12 (16.6%)
Non-determined (15; 10.7%)	2/15 (13.3%)

Note: The 7 years used as an age cut-off was referenced from Hand et al. [17].

**Table 3:** Relationship between demographic, behavioral and cat care variables and presence of *T. gondii* DNA in fecal samples.

Risk factor	PCR *B1* positive	PCR *B1* negative	OR.	95% CI	*p*
Cat age (< 1 year vs. ≥ 1 year)	6 vs. 19	45 vs. 70	0.4	[0.1–1.3]	0.1
Cat sex (male vs. female)	10 vs. 15	66 vs. 49	1.1	[0.4–2.6]	0.8
Food (dry food vs. mix)	14 vs. 11	72 vs. 43	1.3	[0.5–3.1]	0.5
Faucet water for drink (yes vs. no)	22 vs. 3	101 vs. 14	1	[0.2–3.8]	0.9
Cats go outdoors (yes vs. no)	16 vs. 9	61 vs. 54	1.5	[0.6–3.8]	0.3
Hunting activities (yes vs. no)	15 vs. 10	47 vs. 68	2.1	[0.8–5.2]	0.08
Deworming at least once (yes vs. no)	6 vs. 19	87 vs. 28	0.9	[0.3–2.6]	0.9
Stray cats located around the house of the cat owners	24 vs. 1	111 vs. 4	0.8	[0–8]	0.9

### Phylogenetic analysis of *ROP18* sequences

We assayed *ROP18* amplification on *B1* PCR-positive samples and found 6/25 (24%) *ROP18-*positive samples. The Bayesian tree retained (Fig. 1A) clarified the phylogenetic position of *T. gondii* isolates obtained from cats in the present study, relative to the other *T. gondii* strains analyzed. Our sequences clustered into a single clade with Brazilian and Chinese strains (Fig. 1, Clade C). Despite overall support for most of the nodes, we obtained strong support (PP < 0.99) for three major clades: the first clade (A) consisted of Archetype III strains, a second clade (B) grouped the Chinese strains I and II [6] and the Archetype II strains [27], and a third clade (C) consisted of Colombian, Brazilian and Chinese strains (III, according to Gao et al. [17]), and a genotype previously identified and named Archetype I [16]. All six *ROP18* sequences obtained in the present study grouped into clade C, which included *ROP18* sequences from mouse-virulent strains (GT1 and RH) and ocular toxoplasmosis strains (ROP18YM) (Supp. Fig. 1). Major clades were also identified by the unrooted network (Fig. 1B). The haplotype network constructed (Supp. Fig. 2) showed that three *ROP18* sequences obtained from cat feces grouped into a single haplotype, which also contained the Archetype I (RH and GT1) and Chinese III (TgCkjs and TgCtsd1) strains, as well as Brazilian strains. Additionally, the remaining three sequences were found to represent unique haplotypes, separated by one to three mutation steps from the aforementioned haplotype.

## Discussion

Oocyst shedding by the domestic cat (*Felis catus*) is epidemiologically important and shedding needs to be investigated because (1) it is suspected that oocyst density is correlated with high prevalence in some communities, (2) oocysts can lead to outbreaks of acute human toxoplasmosis, and (3) they are probably significantly responsible for infection in animals, which could be animals for human consumption [40]. Likewise, felids are highly susceptible and a single bradyzoite from cyst tissue can be enough to produce infection [9].

In this study, it was not possible to detect *T. gondii* oocysts using the optical microscopy technique in domestic cat feces. The same occurred in an earlier study in Armenia [10]. One possible explanation for the lack of evidence of oocysts in microscopy examination is that, at a given point in time, only 1% of cats could be shedding oocysts [10] and the power of the sample that we calculated could fail to detect such a low prevalence of oocyst shedding. The low frequency of oocysts observed by optical microscopy could also be explained by the fact that we evaluated owned domestic cats that often eat inside houses. The situation could have been different if we had tested wild cats with a natural pray diet. Furthermore, it is important to note that identification may be difficult due to the morphological similarities between *T. gondii* and other coccidian parasites that can be shed in cat feces. Additionally, oocyst shedding by cats occurs over short periods of time, with primary infection mainly in juvenile cats [7, 19]. Other gastrointestinal parasites were found, showing that the frequency of domestic cat feces samples with helminths was lower compared to previous reports in the same city [12]. This could indicate improved deworming habits by cat owners in the city of Armenia.

In contrast with the absence of oocysts via microscopy, PCR detected 17% of cats with the presence of *T. gondii* DNA in cat feces samples. The prevalence of *T. gondii*, according to the DNA frequency, in this study was 17.85% (25/140). This is low in comparison with other studies, like the one performed in Portugal, where 20.5% prevalence was found in domestic cats feces samples using the *B1* sequence in PCR [14]. However, our study found a higher prevalence compared to that observed in a Korean study, where prevalence was 4.5% [20], and a study in Switzerland, where prevalence was 0.4% [4]. This is the first study in Colombia detecting *T. gondii* DNA from cats feces samples, but the frequency of positive samples may be lower compared to previous studies with oocyst prevalence reported at 66.6% [30] and seroprevalence at 84.8%–89.3% [10, 30]. These differences could be associated with the methodology and cats sampled. These PCR results must be interpreted with caution since the *T. gondii* PCR-positives are not always related to oocysts. This is because the presence of *T. gondii* DNA could be due to the presence of bradyzoites of infected prey consumed by a cat [32]. In addition, the PCR methods overestimate exposure to infective parasites because they detect all populations of infectious and non-infectious parasites alive or dead [36]. The utility of PCR detection on cat feces samples is that it offers information on the maximum occurrence and, after sequencing for PCR products, results can be used for genetic analysis of the strains circulating in a geographic region. We did not find statistically significant relationships between the presence of *T. gondii* DNA and the variables obtained from the questionnaire. However, other studies [23, 42] have indicated that cats were probably exposed to the infection from an early age and later, many times throughout their lives, through predation and hunting [43].

Lower prevalence of *T. gondii* in cat feces in some countries could be related to greater access of domestic cats to human food with a low risk of infection [43]. In contrast, high prevalence could be explained by the access of domestic cats to undercooked meat provided by their human owners or by hunting [44].

Previous studies on the genetic diversity of *T. gondii* strains infecting cats in Colombia have shown that parasite populations are highly diverse [10]. Phylogenetic and haplotype analyses including *ROP18* sequences obtained here also found high genetic diversity, despite being obtained from closely located sampling sites. Interestingly, their phylogenetic relationships confirmed the presence of virulent *ROP18* alleles circulating in cats, as reported for patients with ocular toxoplasmosis in the same city [38] (Supp. Fig. 1). However, fine-scale genotyping is imperative to reach definitive conclusions, facilitating the evaluation of the virulence of cat *T. gondii* strains, and their relationships with ocular toxoplasmosis strains. The clades found with recent phylogenetic analyses based on a concatenated matrix that included the *ROP18* gene [15, 33, 34]. In conclusion, this study confirmed a lack of microscopic detection of oocysts in domestic cats in Armenia (Colombia), but that it is possible to detect and analyze genetic diversity by PCR analysis. In this case, the *ROP18* gene was found to be an indicator of virulence of *T. gondii* found in cat feces samples.

## Conflict of interest

The authors declare that they have no conflict of interest.

## Supplementary materials

Supplementary materials are available at https://www.parasite-journal.org/10.1051/parasite/2020023/olm**Supplementary Figure 1.**
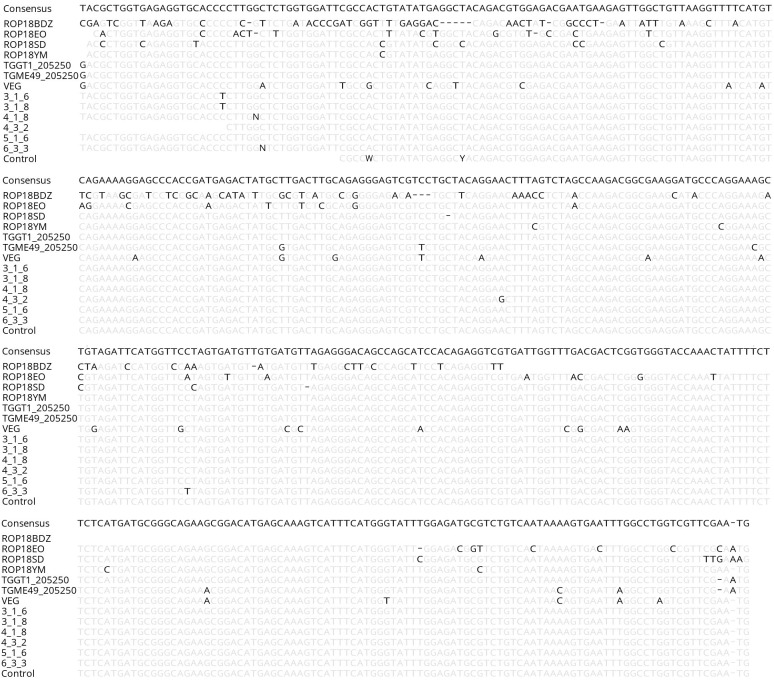
Polymorphic nucleotide sites at the ROP18 polymorphic region for 6 isolates of *T. gondii* from cat feces (plus RH control strain), 4 isolates of *T. gondii* from ocular toxoplasmosis [38], and three sequences representing three archetypal lineages detected for *T. gondii* strains (GT1, ME49 and VEG). Consensus sequence was determined by the percentage of nucleotides common to all sequences. Sites in grey indicate similarity with the consensus sequence.**Supplementary Figure 2.**
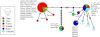
Median-joining network for *T. gondii* haplotypes of the rhoptry protein 18 gene. The size of the circles represents the haplotype frequency and the colors represent the country to which the strain belongs. The number of transversal lines connecting haplotypes represents the number of substitutions between them. Sequences found in cats in the present study are shown with numbers (6_3_3; 5_1_6; 3_1_6; 3_1_8; 4_3_2; 4_1_8).
Supplementary Table 1.Accession numbers and country of *T. gondii* ROP18 gene sequences downloaded from the GenBank database, and later included in our phylogenetic analyses.
