# Supplementary material for: Detection and genotypes of Toxoplasma gondii DNA in feces of domestic cats in Colombia
Source: Parasite. 2020 Apr 17;27:25. doi: 10.1051/parasite/2020023 (PMC7164795; doi:10.1051/parasite/2020023)
Supplement: Supplementary Table 1. — Accession numbers and country of T. gondii ROP18 gene sequences downloaded from the GenBank database, and later included in our phylogenetic analyses. [file parasite-27-25-stabl1.pdf]

Name	Accession	Country
ARI	JX045322	USA
B41	JX045323	USA
CAST	JX045345	USA
COUG	JX045321	Canada
ENT	JX045328	France
FOU	JX045332	France
GT1	JX045329	USA
GT1.1	GQ243202	USA
M7741	JX045353	USA
MAS	JX045339	France
MAS.1	GQ243203	Brazil
ME49	JX045319	USA
MOR	JX045326	France
p89	JX045347	USA
PIH	JX045320	USA
PTG	GQ243204	USA
QHO	GQ243205	China
RH	JX045330	USA
ROP18BDZ	JN579944	Colombia
ROP18EO	JN579943	Colombia
ROP18SD	JN579945	Colombia
ROP18YM	JN579946	Colombia
RUB	JX045336	France
T61	JX045350	USA
TgCatBr1	JX045340	Brazil
TgCatBr2	JX045333	Brazil
TgCatBr3	JX045349	Brazil
TgCatBr5	GQ243206	Brazil
TgCatBr6	JX045342	Brazil
TgCatBr9	JX045341	Brazil
TgCatBr10	JX045343	Brazil
TgCatBr18	JX045344	Brazil
TgCatPRC2	KQ983279	China
TgCgCa1	GQ243207	Canada
TgCkjs	KY628208	China
TgCtbj	KY628207	China
TgCtgd1	KY628196	China
TgCtgd2	KY628197	China
TgCtGZ1	GQ243209	China
TgCtGZ2	GQ243210	China

TgCtGZ3	GQ243211	China
TgCtGZ4	GQ243212	China
TgCtGZ5	GQ243213	China
TgCtGZ6	GQ243214	China
TgCtGZ7	GQ243215	China
TgCtGZ8	GQ243216	China
TgCtsd1	KY628200	China
TgCtsd3	KY628201	China
TgCtsd4	KY628202	China
TgCtsd5	KY628203	China
TgCtsx1	KY628198	China
TgCtsx2	KY628199	China
TgCtwh3	KY628204	China
TgCtwh5	KY628205	China
TgCtwh6	KY628206	China
TgCtxz3	KY628209	China
TgCtxz5	KY628210	China
TgCtxz7	KY628211	China
TgCtxz8	KY628212	China
TgPgPYS	GQ243208	China
VAND	JX045337	France
VEG	JX045348	USA
